# Chemokine (C-X-C motif) receptor 4 RNA interference inhibits bone metastasis in breast cancer

**DOI:** 10.3892/ol.2014.2096

**Published:** 2014-04-28

**Authors:** HENG ZENG, WEI WEI, XIAOTAO XU

**Affiliations:** 1 Department of Orthopedics, Tongji Hospital, Tongji Medical College, Huazhong University of Science and Technology, Wuhan, Hubei 430030, P.R. China; 2 Department of Trauma and Microsurgery, 324 Hospital of PLA, Chongqing 400020, P.R. China; 3 Department of Oncology, Renmin Hospital, Wuhan University, Wuhan, Hubei 430060, P.R. China

**Keywords:** chemokine (C-X-C motif) receptor 4, breast cancer, bone metastasis, phosphoinositide 3-kinase/protein kinase B, matrix metalloproteinase-9

## Abstract

Chemokine (C-X-C motif) receptor 4 (CXCR4) has been found to closely correlate with the incidence, development, treatment and prognosis of breast cancer. The aim of the present study was to investigate the effects of CXCR4 on bone metastasis in breast cancer and to explore the mechanisms of this process. CXCR4 small interfering RNA was transfected into the breast cancer cell line, MDA-MB-231BA-rfp, and the cell proliferation and invasion abilities of the cells were measured using cell counting kit-8 cell proliferation and Transwell assays. A mouse model of breast cancer with bone metastasis was prepared and the bone metastasis was confirmed using micro-positron emission tomography. The associated proteins were detected by western blot analysis and the results showed that CXCR4 RNAi inhibited the cell proliferation and invasion ability of the MDA-MB-231BA-rfp cells. In addition, CXCR4 RNAi inhibited the duration and extent of bone metastasis in the MDA-MB-231BA-rfp cells in the mouse model, while the inhibition of CXCR4 RNAi blocked the phosphatidylinositide 3-kinase (PI3K)/protein kinase B (AKT)/matrix metalloproteinase (MMP)-9 pathway. In conclusion, the present study demonstrated that CXCR4 RNAi inhibits bone metastasis and the cell proliferation and invasion abilities of breast cancer cells. Furthermore, the CXCR4/PI3K/AKT/MMP-9 pathway may be important in the bone metastasis of breast cancer.

## Introduction

At present, breast cancer is the most common malignant tumor in females, and the bone metastasis of breast cancer has a direct effect on patient prognosis, which results in a marked increase in the mortality rate (1). The molecular mechanisms of metastasis remain unclear, as the factors involved in the metastatic process are complicated. However, a recent study has revealed that chemokine (C-X-C motif) receptor 4 (CXCR4) closely correlates with the incidence, development, treatment and prognosis of breast cancer (2–6). In the present study, the targeted downregulation of CXCR4 expression in the MDA-MB-231BA-rfp breast cancer cell line (with a high propensity to metastasize to bone) via RNA interference (RNAi) techniques was performed to analyze the effect of CXCR4 on the ability of cancerous cells to metastasize to the bone, as well as to investigate the underlying mechanisms. The results offer an enhanced understanding of the molecular mechanisms that lead to breast cancer bone metastasis.

## Materials and methods

### Experimental materials

The human breast cancer cell line, MDA-MB-231BA-rfp, was stored frozen in liquid nitrogen. Dulbecco’s modified Eagle’s medium with 10% fetal calf serum was purchased from Gibco-BRL (Carlsbad, CA, USA). The rabbit polyclonal antibodies against human CXCR4, phosphatidylinositide 3-kinase (PI3K), protein kinase B (AKT), and matrix metalloproteinase (MMP)-9 were purchased from Santa Cruz Biotechnology, Inc. (Santa Cruz, CA, USA). The western blot analysis kits were purchased from Wuhan Boster Biological Technology, Ltd. (Wuhan, China). This study was approved by the Institutional Review Board of Tonji Hospital, Huazhong University of Science and Technology (Wuhan, China).

### CXCR4 small interfering RNA (siRNA) construction and transfection

The two CXCR4 siRNA oligonucleotide sequences purchased from Dharmacon, Inc., (Lafayelle, CA, USA) were identified and matched with the following CXCR4 cDNA sequences obtained from GeneBank through a BLAST search: Sense, 5′-UAAAAUCUUCCUGCCCACCdTdT-3′ for siRNA1; and sense, 5′-GGAAGCUGUUGGCUGAAAAdT dT-3′ for siRNA2. In addition, a negative control siRNA sequence was formulated and synthesized, as follows: 5′-UUCUCCGAACGUGUCACGUUUGUGC-3′. The MDA-MB-231BA-rfp cells (1×10^5^ cells/ml) were then transfected with 100 nM CXCR4 siRNA mediated by oligofectamine (Invitrogen Life Technologies, Carlsbad, CA, USA). The following groupings were then determined: Con-B group, blank control group; Con-A group, empty vector group; S1 group, siRNA1 transfection group; S2 group, siRNA2 transfection group; and Sn group, negative control siRNA sequence transfection group. With the exception of isocyanic phosphate-buffered saline therapy for Con-B group and isocyatic oligofectamine therapy for Con-A group, the follow-up procedures for the five groups were the same. The siRNA sequence that exhibited the highest interfering efficiency was then selected to continue the study.

### Western blot analysis

The MDA-MB-231BA-rfp cells in the exponential growth phase were centrifuged at 30,000 × g at 4°C for 5 min to separate the supernatant from the cellular debris (centrifugation radius, 4 cm) following the application of radioimmunoprecipitation assay protein lysis buffer (Wuhan Boster Biological Technology, Ltd.). Next, the level of protein expression was determined using the bicinchoninic acid assay method. Subsequently, 50 μg of protein was harvested and 2× loading buffer was added to the protein samples, which were then were then heated to 100°C for 5 min. Next, following SDS-PAGE separation, the samples were loaded onto a nitrocellulose filter and then combined with the specific antibodies and corresponding diantibodies. Finally, the samples were stained using enhanced chemiluminescence kit (Wuhan Boster Biological Technology, Ltd.) prior to the X-ray films being exposed, developed and fixed. Gray scale images were also captured and analyzed using BandScan software (Glyko, Novato, CA, USA).

### Cell invasion assay in vitro

Transwell chamber models (Chemicon, Temecula, CA, USA) were performed to prepare a cell suspension containing 1×10^5^ cells/ml, of which 50 μl was added to the upper chamber. At 24 h post-incubation, the cells located on the inner layer of the chamber were removed and the remaining cells were fixed using 10% formalin. Giemsa stain was used to count the number of invasive cells that had migrated through the membrane.

### Cell counting kit (CCK)-8 cell proliferation assay

The cells were digested with 0.25% pancreatic enzyme (Wuhan Boster Biological Technology, Ltd.), which resulted in a cell suspension containing 1.2×10^4^ cells/ml. Next, the cell suspension was seeded into 96-well plates (200 μl per well) and separated into the following three groups: Control group, lipopolysaccharide (1.0 μg/ml) intervention group and Toll-like receptor-4 intervention group. After 24 h, CCK-8 (10 μl/well) was added and the cells were incubated for another 2 h. The absorbance at 450 nm was measured using a microplate reader and the proliferation ability of the mesenchymal stem cells of different mice were analyzed.

### Tumorigenesis in nude mice

In total, 24 C57BL/6 nude mice were kept in a biologically clean animal laboratory at a temperature of 23±1°C, with a relative humidity of 55–60%. The mice were housed six per cage in polycarbonate cages (8×13.5×8.1 cm in size). The dry sawdust bedding was sterilized and replaced every five days, and sterile distilled drinking water was provided. Animals were randomized into intervention (n=12) and control (n=12) groups following one week of acclimation to the same conditions. MDA-MB-231BA-rfp cells in the exponential growth phase were dissociated to prepare a single cell suspension, of which the cell density was adjusted to 1×10^6^ cells/ml, with a cell viability of >95%. Briefly, 2 ml of the cell suspension was injected into the caudal veins of the mice. The suspension included MDA-MB-231BA-rfp cells transfected with CXCR4 siRNA for the intervention group and MDA-MB-231BA-rfp cells without CXCR4 siRNA transfection for the control group.

### Micro-positron emission tomography (PET) detection of bone metastasis in nude mice

The mice were deprived of food and water for 8 h and anesthetized via inhalation of 2% isoflurane prior to microPET. At 40 min after the injection of the radioactive tracer, fludeoxyglucose, into the tail vein, the mice were placed in a prone position and imaging was performed for 10 min. Bone metastasis was analyzed using ASIPro (Siemens Medical Solutions USA, Inc., Knoxville, TN, USA) to determine the region of interest (ROI), and the maximum standard uptake value (SUV) was used for the statistical analysis. The ROI was evaluated by a researcher blinded to the experimental schedule and groupings.

### Statistical analysis

All data are presented as the mean ± standard deviation and were analyzed using SPSS version 16.0 (SPSS, Inc., Chicago, IL, USA) by two-tailed t-test. P<0.05 was considered to indicate a statistically significant difference.

## Results

### Interference effects of CXCR4 siRNA

**Figure 1:**
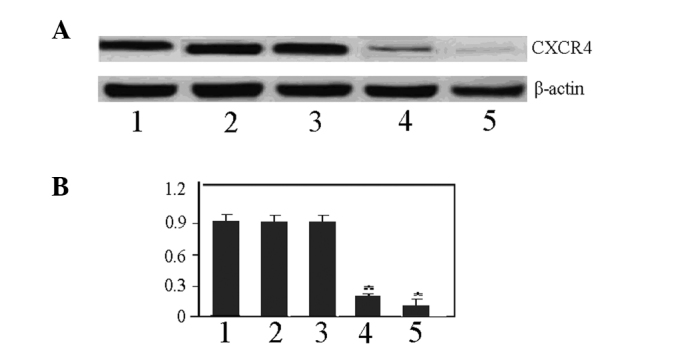
CXCR4 siRNA reduces the expression of CXCR4. (A) SDS-PAGE and (B) the gray scale ratio of each band: Lane 1, blank control; 2, empty vector; 3, Sn transfection; 4, siRNA1 transfection; and 5, siRNA2 transfection groups. CXCR4, chemokine (C-X-C motif) receptor 4; siRNA, small interfering RNA.

The results of the western blot analysis revealed that the expression of CXCR4 in the MDA-MB-231BA-rfp cell line was significantly downregulated in the S1 and S2 groups compared with the Con-B, Con-A and Sn groups at 24 h after the transfection with 100 nM CXCR4 siRNA. The interference efficiency was calculated using the following formula: Interference efficiency = (downregulation range of CXCR4 in control group - downregulation range of CXCR4 in intervention group) / downregulation range in control group. The results showed interference efficiencies of 83 and 92% in the S1 and S2 groups, respectively. Therefore, since the S2 group exhibited a relatively higher interference efficacy than the S1 group, the S2 group was selected to be used as the CXCR4-specific interference sequence for the sequential study (Fig. 1).

### CXCR4 siRNA inhibition of cellular proliferation and invasion

**Figure 2:**
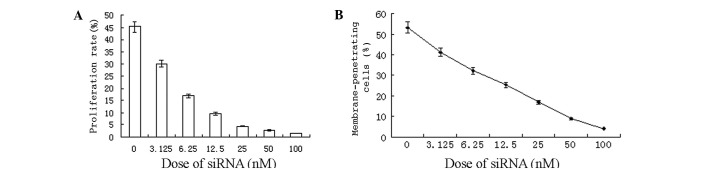
CXCR4 siRNA inhibits cellular proliferation and invasion. (A) The cell counting kit-8 (CCK-8) cell proliferation assay revealed that the proliferation rate in the siRNA2-transfected cells decreased with increasing concentrations of transfection reagent. (B) The Transwell migration assay indicated that the number of cancer cells that migrated through the filter membrane substantially decreased with increasing siRNA concentration. CXCR4, chemokine (C-X-C motif) receptor 4; siRNA, small interfering RNA.

Based on the finding that the S2 group was found to be the most effective in reducing CXCR4 expression, S2 was selected as the CXCR4-specific sequence to proceed with in the study. The CCK-8 proliferation assay revealed that the proliferation rate of the S2-transfected cells was decreased with increasing concentrations of the transfection reagent (0, 3.125, 6.25, 12.5, 25, 50 and 100 nM; Fig. 2A). At 48 h after the transfection of the breast cancer cells with the varying S2 concentrations, the results of the Transwell migration assay also indicated that the number of cancer cells that had migrated through the filter membrane was substantially decreased with increasing siRNA concentration (Fig. 2B).

### CXCR4 RNAi inhibition of bone metastasis in nude mice

**Figure 3:**
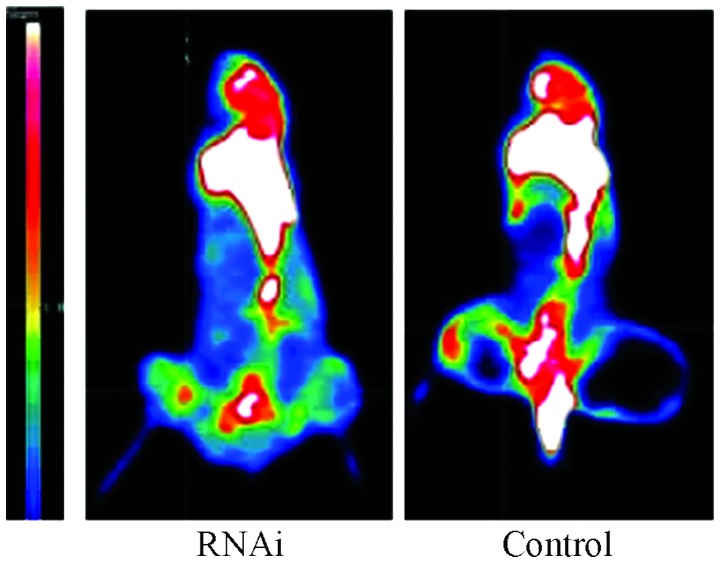
Micro-positron emission tomography (PET) images of the two groups, with a color code indicating the glucose metabolism levels; red indicates high levels and blue indicates low levels (radioactive tracer, ^18^F-FDG). The bright white region is positively proportional to the uptake volume of ^18^F-FDG by the tumors in the region. The direct viewing of images demonstrated that the bright white regions in the lower extremities, ribs and spine were considerably larger in the control groups than in the interference group. ^18^F-FDG, ^18^F-fludeoxyglucose; RNAi, RNA interference.

The nude mice in the intervention group were injected with MDA-MB-231BA-rfp cells that had been transfected with 100 nM S2 for 48 h (n=8), and the control group were injected with an equal amount of non-transfected MDA-MB-231BA-rfp cells (n=8). MicroPET analysis revealed that the nude mice in the control group exhibited a mild breakdown of cortical bone in the lower extremities four weeks after tumor cell injection. By contrast, it was not until the sixth week after tumor cell injection that distinct bone invasion, indicating the emergence of bone metastasis, was identified in the interference group. Direct observation of the microPET images six weeks after the injections revealed that the bright white region in the lower extremities, ribs and spine was much larger in the control group than in the interference group (Fig. 3). Semi-quantitative analysis was performed based on the SUV ratios, which revealed that the SUVmax in the interference group was 9.38±0.54 versus 2.13±0.21 in the control group (P<0.01), indicating that the onset and degree of MDA-MB-231SA-rfp cell bone metastasis may be significantly inhibited by CXCR4 RNAi.

### CXCR4 RNAi downregulates MMP-9 via blockade of the PI3K/AKT signaling pathway

**Figure 4:**
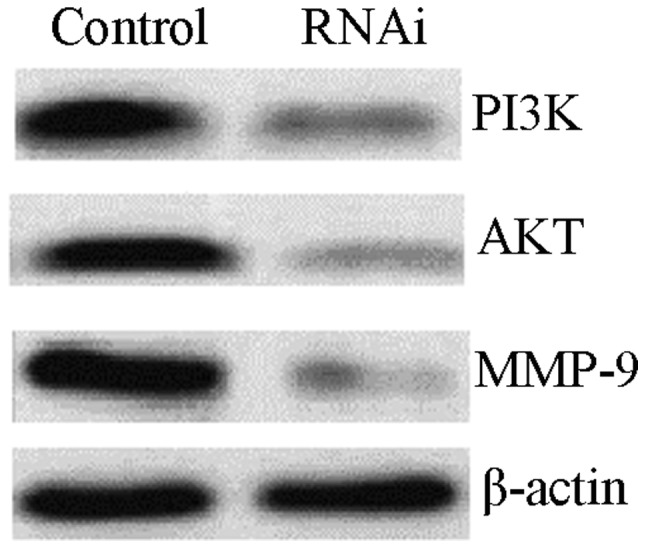
Western blot analysis revealed that CXCR4 RNAi inhibited PI3K/AKT signaling and the expression of MMP-9. PI3K, phosphatidylinositide 3-kinase; AKT, protein kinase B; MMP-9, matrix metalloproteinase-9; RNAi, RNA interference.

To investigate the potential mechanism of CXCR4 RNAi controlling breast cancer metastasis to the bone, western blot analysis was used to analyze the expression of PI3K/AKT/MMP-9 following the silencing of CXCR4 by RNAi. As a result, the expression of PI3K/AKT/MMP-9 was reduced by CXCR4 RNAi, with the levels of PI3K, AKT and MMP-9 far lower than those in the control group (0.32±0.06 vs. 0.89±0.12 for PI3K; 0.16±0.03 vs. 0.86±0.10 for AKT; and 0.12±0.02 vs. 1.12±0.16 for MMP-9; P<0.01; Fig. 4).

## Discussion

CXCR4 is a highly conserved G protein-coupled receptor of the chemokine receptor family that mediates chemotactic activity and is a receptor specific to CXC ligand 12 (CXCL12) (7). Previous studies have demonstrated that CXCR4 is the most common chemokine receptor expressed in tumor cells, and that it plays a predominant role in the migration and invasion of tumors (8–11). In addition, a recent study has revealed that CXCR4 is vital for the migration, invasion, treatment and prognosis of breast cancer (12,13). Furthermore, Gil *et al* (14) reported that a virus-coated CXCR4 antagonist is effective in the treatment of primary or metastatic breast cancer, functioning by disrupting the internal environment for tumor cell growth and inhibiting the vascularization and expression of CXCL12 and vascular endothelial growth factor (VEGF). Additionally, Ling *et al* (15) reported that the CXCR4 antagonist, AMD3465, inhibits the growth and migration of breast cancer by partially blocking signal transducer and activator of transcription 3 signaling, which has an impact on tumor and immune cells in the internal tumor environment.

In the present study, the effect of CXCR4 on the bone metastasis of breast cancer by targeting the downregulation of CXCR4 using RNAi techniques was observed (Fig. 1). Firstly, the CCK-8 cell proliferation and Transwell chamber assays were used to detect the oncological characteristics of the breast cancer cells prior to and following CXCR4 suppression. The observations revealed that CXCR4 siRNA significantly inhibits the proliferation and invasion of breast cancer cells (Fig. 2). Therefore, CXCR4 is key in the growth and proliferation of breast cancer cells, indicating that the control of its activity may significantly reduce the proliferation of breast cancer cells *in vitro*. In addition, as CXCR4 is involved in the motility and chemotaxis of breast cancer cells, the suppression of CXCR4 expression may significantly reduce the migration of breast cancer cells to distant organs (16). Furthermore, Wendel *et al* (17) reported that CXCR4/CXCL12 is significant in the migration of breast cancer cells by affecting the adhesiveness, morphology and migration of the cells and the regulation of the expression of the protein family in the extracellular matrix.

Based on the results of the *in vitro* experiment, an *in vivo* experiment was conducted to investigate the effect of CXCR4 inhibition on breast cancer bone metastasis. The mouse model of breast cancer was simulated by injecting MDA-MB-231BA-rfp cells transfected with CXCR4 RNAi into the tail vein. As a result, the onset of the bone metastasis of breast cancer cells was prolonged and the metastasis was attenuated with the interference of CXCR4, which tentatively confirmed that CXCR4 RNAi inhibits the spread of breast cancer cells to the bone. A previous cohort study indicated that the detection of CXCR4 expression is of great value in predicting the bone metastasis of breast cancer (18). To exclude non-bone metastasis, the present study used MDA-MB-231BA-rfp cells, which are breast cancer cells with a high bone-specific metastatic potential, in order to establish the bone metastasis model.

The mechanism of breast cancer cell metastasis to the bone is complicated, however, the CXCR4/stromal cell-derived factor-1 axis has a vital regulatory function (19). To study the effect of CXCR4 in the regulation of the PI3K/AKT signaling pathway, CXCR4 was inhibited in the current study. As a result, the inhibition of CXCR4 had an impact on the activity of PI3K/AKT. Ping *et al* (20) also reported that the vascularization of glioma cells is attenuated by the downregulation of CXCR4 using the CXCR4 antagonist, AMD3100, or RNAi, and the reduction of VEGF expression via the inhibition of the PI3K/AKT signaling pathway. However, Zheng *et al* (21) reported that CXCR4 mediates the endothelial progenitor cells via the PI3K/AKT signaling pathway. To further investigate the effect of CXCR4 on the regulation of downstream cytokines, its impact on the expression of MMP-9 was observed in the present study. The results showed that the expression of MMP-9 was reduced with the interference of CXCR4. Consequently, we hypothesize that the CXCR4/PI3K/AKT/MMP-9 signaling pathway is involved in the bone metastasis of breast cancer.

In conclusion, the preliminary *in vitro* experiment revealed that the proliferation and invasion of breast cancer cells is inhibited with the interference of CXCR4. The construction of *in vivo* models of breast cancer metastasis to the bone further confirmed the inhibition of bone metastasis as a result of CXCR4 interference and the involvement of CXCR4/PI3K/AKT/MMP-9 signaling in bone metastasis. The present study provides supporting evidence for the mechanism of the metastasis of breast cancer to the bone.
